# Thimerosal-Derived Ethylmercury Is a Mitochondrial Toxin in Human Astrocytes: Possible Role of Fenton Chemistry in the Oxidation and Breakage of mtDNA

**DOI:** 10.1155/2012/373678

**Published:** 2012-06-28

**Authors:** Martyn A. Sharpe, Andrew D. Livingston, David S. Baskin

**Affiliations:** Department of Neurosurgery, The Methodist Hospital, 6565 Fannin Street, Houston, TX 77030, USA

## Abstract

Thimerosal generates ethylmercury in aqueous solution and is widely used as preservative. We have investigated the toxicology of Thimerosal in normal human astrocytes, paying particular attention to mitochondrial function and the generation of specific oxidants. We find that ethylmercury not only inhibits mitochondrial respiration leading to a drop in the steady state membrane potential, but also concurrent with these phenomena increases the formation of superoxide, hydrogen peroxide, and Fenton/Haber-Weiss generated hydroxyl radical. These oxidants increase the levels of cellular aldehyde/ketones. Additionally, we find a five-fold increase in the levels of oxidant damaged mitochondrial DNA bases and increases in the levels of mtDNA nicks and blunt-ended breaks. Highly damaged mitochondria are characterized by having very low membrane potentials, increased superoxide/hydrogen peroxide production, and extensively damaged mtDNA and proteins. These mitochondria appear to have undergone a permeability transition, an observation supported by the five-fold increase in Caspase-3 activity observed after Thimerosal treatment.

## 1. Introduction

### 1.1. Thimerosal and Ethylmercury

Thimerosal is a preservative that is widely used in medical products, including as a preservative in vaccines, immunoglobulin preparations, skin test antigens, antivenins, ophthalmic and nasal products, and tattoo inks, and is composed of 49.6 percent ethylmercury by weight [1]. The widespread use of Thimerosal exposes many to its potential toxic effects, especially *in* *utero* and in neonates. We report the results of a series of experiments using cultured normal human astrocytes (NHA) exposed to Thimerosal to study the compound's effect on astrocyte mitochondria.

### 1.2. Oxidative Stress and Brain

The brain utilizes 20% of the oxygen consumed by the body but constitutes only 2% of the body's mass [2]. Some 5% of molecular oxygen consumption may arise from its reduction to superoxide [3]. The majority of superoxide generated in cells comes from the reaction of molecular oxygen with flavin or quinone radicals, which are partly generated during respiration within complexes of the mitochondrial respiratory chain [4]. The rate of reactive oxygen species (ROS) production increases steeply with increased mitochondrial membrane potential [3]. Superoxide has a very short half-life in cells as it is rapidly dismutased by either the cytosolic Cu-Zn superoxide dismutase (SOD) or the Mn-SOD in the mitochondrial matrix, producing molecular oxygen and hydrogen peroxide. Thus, generation of superoxide is always accompanied by hydrogen peroxide production, and so opens up the possibility of hydroxyl radical (HO^•^) generation via Fenton/Haber-Weiss chemistry [5]. Fenton metals, including iron and copper, catalyze the production of HO^•^ from superoxide/hydrogen peroxide and so the free, unchelated levels of transition metals inside cells are very low and normally all stored in an oxidized state. Normally, these metals are tightly bound to various metallochaperones, such as the ferric iron chelator ferritin.

### 1.3. Astrocytic Antioxidants in Humans

Astrocytes are the major supporting cells of the brain and one of their key features is their ability to become “reactive” towards infectious agents and use chemical warfare, upregulating iNOS to generate high levels of nitric oxide and NADPH oxidase to generate superoxide, hydrogen peroxide, peroxynitrite, and other oxidative per-species (see [6] and references within). The types and levels of antioxidant enzymes of NHA are rather different from most other cell types and the levels of different enzymatic antioxidant enzyme change when NHA transition from “unreactive” to “reactive” states. In many cell types the main defense against peroxide stress are selenol containing enzymes including the glutathione peroxidases (GPx) and thioredoxin reductase (TrxR). GPx is not present in detectable levels in human “unreactive” astrocytes in normal brain [7] and it appears that GPx is only present in high levels in “reactive” astrocytes [8, 9]. TrxR levels in normal human brain are also low, but is significantly elevated in the brains of Alzheimer's patients, especially at the site of amyloid plaques where “reactive” astrocytes are present [10]. It has been shown that in cultured NHA that TrxR expression is under tight regulation, with increases from very low basal levels, under the control of cytokines and growth factors [11].

Peroxiredoxins, including the mitochondrial Peroxiredoxin V, are an important class of peroxide/peroxynitrite detoxification enzymes that are sensitive to organomercury [12]. Like the selenol-based antioxidant enzymes, these thiol-based antioxidant proteins are only found in very low levels in human astrocytes [13].

There is much evidence to suggest that catalase, rather than cysteine or selenocysteine-based peroxidases, is the main enzymatic peroxidase in “unreactive” NHA [14]. NHA also have high levels of reduced glutathione (GSH), capable of detoxifying peroxides via direct chemistry, and high levels of all three superoxide dismutases [15, 16]. Catalase and the manganese superoxide dismutase are both upregulated when astrocytes are subjected to oxidative stress [14, 16]. In cell types where selenol/thiol containing peroxidases are the major enzymes that detoxify peroxides, organomercury toxicity tends to result from loss of antioxidant enzyme function coupled with an increase in the rate of oxidant production [17, 18].

There is a large literature examining the role of organomercury toxicity and the involvement of selenoenzymes TrxR and glutathione peroxidase, GPx, see [18] and references therein; however, these data may not apply to NHA, especially “unreactive” NHA which appear not to make extensive use of these organomercury sensitive detoxification enzymes.

### 1.4. Localization of Organomercury-Induced Damage

Ethylmercury is a lipophilic cation which can cross the blood-brain barrier [19–22]. The octanol/water partition coefficients of methyl and ethylmercury are 1.4 to 1.8 [21, 23], at intracellular pH and [Cl^−^], thus both organomercury compounds will predominately exist as lipophilic cations inside cells. Mitchell demonstrated that lipophilic cations accumulate inside mitochondria, in a Nernstian fashion, driven by the steady state membrane potential [24]. Given that the typical mitochondrial membrane potential of astrocytes and neurons is between 140–170 mV [25], one would, *a prior*, expect the concentration of these organomercury compounds within mitochondria to be approximately 1000 times greater than the cytosolic concentration.

### 1.5. Ethylmercury and Mitochondria

We postulate that this compound is preferentially taken up into the mitochondria of NHA causing damage to the respiratory chain and subsequent ROS production. The damage of a cell's mitochondria leads to the activation of the apoptotic cascade and subsequent cell death [3, 4, 24, 26–31]. This may be clinically relevant in the setting of a patient who harbors a known or unknown mitochondrial disorder. In the setting of a mitochondrial disorder, a specific mitochondrial toxin could be life altering or life threatening.

We designed this series of experiments to examine the effects of Thimerosal-derived ethylmercury on human astrocyte apoptosis by choosing a time course of cell examination after treatment that would showcase the early stages of apoptosis. We proposed that by examining the cells in an early phase, sixty minutes after ethylmercury dosing, we could visualize the compound's effect on the mitochondria and mitochondrial DNA (mtDNA).

## 2. Methods

Normal human astrocytes (NHA) were obtained from Lonza (Walkersville, MD, USA) and grown subject to their recommendations. NHA were grown to confluency in Astrocyte Cell Basal Medium supplemented with 3% FBS, Glutamine, Insulin, fhEGF, GA-1000 and Ascorbic acid in 16-well Lab-Tek slide chambers (Nalge Nunc, Rochester, NY, USA), in a total volume of 240 *μ*l.

### 2.1. Probes in Living Cells

NHA were incubated for 1 hour with probes before fixation. Fixation in buffered paraformaldehyde (PFA) was performed in two stages. Firstly, a 50 *μ*l aliquot of ice-cold 8% PFA was added to each well, then gently aspirated and the wells were twice washed with ice-cold 2% PFA and then allowed to completely fix at 4°C. After fixation cells were washed twice in x1 PBS (Thermo Fisher Scientific, Rockford, IL, USA). The tanks were then removed from the slides, the well area covered with Fluoromount-G (SouthernBiotech, IL, USA), cover-slipped and sealed with nail varnish.

DNA was visualized using 1 *μ*M Hoechst 33258 (Cat no. H1398), mitochondrial membrane potential with 500 nM Mitotracker Red [27] (Cat no. M22425), hydrogen peroxide using 5 *μ*M H_2_DCFAM [31, 32] (Cat no. D399), mitochondrial superoxide generation with 5 *μ*M MitoSOX Red [26] (Cat no. M36008); HO^•^ was assayed using 5 *μ*M hydroxyphenyl fluorescein [32] (HPF) (Cat no. H36004), with reagents obtained from Molecular Probes (Eugene, OR, USA).

### 2.2. Probes in Fixed Cells

Fixed cells were permeabilized using x1 PBS with 0.1% Triton X-100. Hydrazine reactive aldehyde/ketones were labeled using 225 *μ*M Biotin-XX Hydrazide [33] (Cat no. B2600) and visualized using Texas Red Avidin (Cat no. A820).

The activity of Caspase-3 in fixed, 0.1% Triton permeabilized cells was measured using the Molecular Probes R110-EnzChek Assay Kit (Cat no. E13184), incubating cells for 1 h at 37°C [34].

### 2.3. DNA Labels

The measurements and quantification of DNA 3′OH (*dd*TUNEL), oxidized DNA bases (*Fpg*-*dd*TUNEL) and blunt ended breaks by use of the *dd*TUNEL and blunt-ended ligation were performed as described in our recent publications [35, 36]. Biotinylated *dd*UTP and biotinylated blunt ended oligonucleotide probe were visualized using Molecular Probes FITC labeled avidin (Cat no. 434411).

### 2.4. *dd*TUNEL

A *Tdt* reaction buffer was prepared daily diluting a stock solution 1 : 5 of TUNEL buffer (125 mM Tris-HCl, 1 M sodium cacodylate, 1.25 mg/mL BSA, pH 6.6) and a 25 mM cobalt chloride stock solution, 1 : 25. Each well was twice washed in this reaction buffer and then incubated with 50 *μ*l of reaction buffer containing 20 units/mL of *Tdt* and 250 nM of Biotin-16-*dd*UTP (Roche, IN, USA). Labels were developed using labeled FITC-avidin (Cat no. 434411).

### 2.5. *CIAP*-*dd*TUNEL

Each sample, having previously undergone *dd*TUNEL, was washed and incubated with NE Buffer 3 for 30 minutes and then with ≈50 *μ*l of the same buffer containing 100 units/mL of calf intestinal alkaline phosphatases (Sigma) for ≥2 hours and the newly generated, 3′PO_4_→3′OH, ends.

### 2.6. *Fpg-dd*TUNEL Assay

Following *dd*TUNEL/*CIAP*-*dd*TUNEL, capping all 3′OH/3′PO_4_ ends with authentic, unlabeled avidin, samples were washed twice in 10 mM HEPES, 10 mM NaCl, 2 mM EDTA and 0.1% BSA and then 50 *μ*l of the same buffer containing 100 units/mL of formamidopyrimidine DNA glycosylase (*Fpg*) (USB, Cleveland, OH, USA) was applied to each of the wells, then incubated in a humidified box ≥2 hours. Each sample was washed twice in x1 PBS (Thermo Fisher Scientific, Rockford, IL, USA) twice in NEBuffer 3 and ≈50 *μ*l of the same buffer containing 100 units/mL of *CIAP* was applied to each section and incubated for ≥2 hours; samples then underwent a third round of *dd*TUNEL and labeling with FITC-avidin.

### 2.7. Blunt-Ended DNA Breaks

We used a biotinylated version of the blunt-ended oligonuleotide probe previously described [36]. The wells were preincubated in the ligation buffer without the probe (66 mM-Tris HCl, pH 7.5, 5 mM MgCl_2_, 0.1 mM dithioerythritol, 1 mM ATP, and 15% polyethylene glycol-8000) to ensure even saturation. The buffer was aspirated, and the full ligation mix containing the ligation buffer with probe, 35 *μ*g/*μ*l, and 0.5 U/*μ*l T4 DNA ligase (New England Biolabs, Ipswich, MA, USA) was applied to the sections, which were then incubated in a humidified box overnight.

### 2.8. Thimerosal

**Figure 1:**
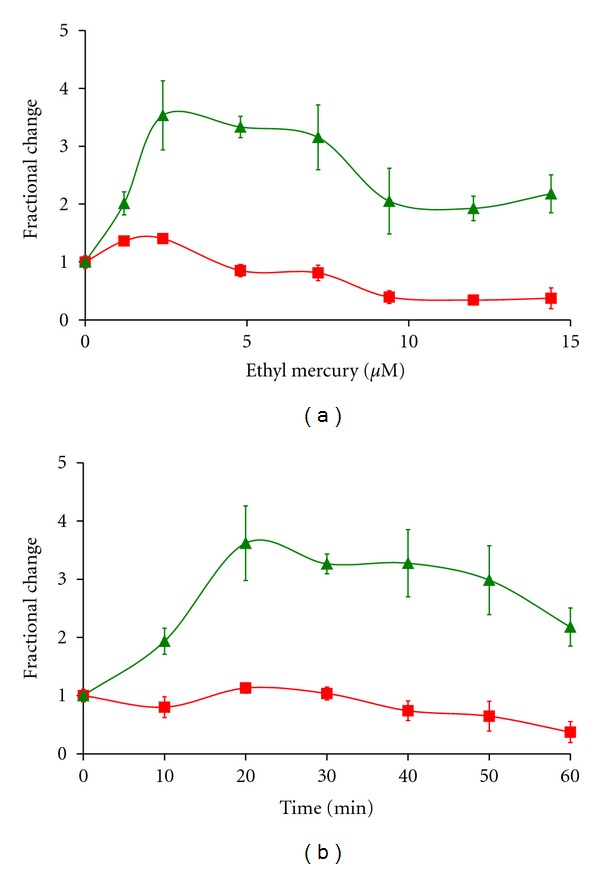
Changes in mitochondrial membrane potential and of ROS generation induced by ethylmercury in Normal Human Astrocytes. Changes in mitotracker and ROS induced DCF levels caused by incubation with increasing concentrations of Thimerosal (b) and the time course of incubation with 14.4 *μ*M Thimerosal (a), with respect to control cultures. Cells were imaged in the centerfield of three independent wells, consisting of an average of 44 cells per field, with a standard deviation of 16.5.

Thimerosal ≥97% (HPLC) and all unspecified regeants were obtained from Sigma-Aldrich (St. Louis, MO, USA), unless otherwise specified. Thimerosal solutions were prepared in x1 PBS (Thermo Fisher Scientific, Rockford, IL, USA) to a maximum concentration of 360 *μ*M and 10 *μ*l were added to the 240 *μ*l astrocytic volume. 3% FCS was present in the NHA media throughout the time course. To generate the time course shown in Figure 1, NHA were exposed to Mitotracker, H_2_DCFAM and Hoechst at *t* = 0. Additions of 10 *μ*l aliquots were added at 10 minute intervals, to different wells in sequence, so that all the cells had the same length of exposure to the reporters, but different temporal exposure to Thimerosal.

### 2.9. Epifluorescence Microscopy

The signal was acquired using a Nikon Eclipse TE2000-E fluorescent microscope equipped with a CoolSnap ES digital camera system (RoperScientific) containing a CCD-1300-Y/HS 1392 × 1040 imaging array cooled by a Peltier device.

Images were recorded and analyzed using Nikon NIS-Elements software and images were stored as both  .jpeg200 and  .jpg files.

## 3. Results

### 3.1. Changes in Mitochondrial Membrane Potential and ROS Generation Following Thimerosal Incubation

The effect of ethylmercury on the fluorescence levels of the three reporters was investigated in two ways. The concentration dependence of ethylmercury towards NHA was studied by adding to 0–14.4 *μ*M Thimerosal to the cell media at *t* = 0. In addition, we investigated the temporal changes caused by the addition of 14.4 *μ*M Thimerosal at *t* = 0, 10, 20, 30, 40, and 50 minutes before fixation at 60 minutes. We imaged the center field of three independent wells, at each time point or concentration, collecting the fluorescence levels of the three reporters of an average of 44 ± 18 individual astrocytes per center field. In Figure 1 we show the changes in the levels of MT and ROS (via DCF formation) as a function of Thimerosal concentration (Figure 1(a)) and of changes induced by incubation with 14.4 *μ*M Thimerosal over time.

It can be seen that low concentrations of ethylmercury cause an increase in both signals. The finding that ethylmercury increases ROS generation is not surprising, given the well-known effects this agent has in disrupting cellular thiol/glutathione-based antioxidant defenses [20, 22, 30]. The hyperpolarization of mitochondrial membrane potential was unexpected, given that depolarization of mitochondria has been observed in most cell types prior to apoptosis. At higher concentrations (>7.2 *μ*M Thimerosal) a loss of mitochondrial signal and of DCF is observed. This loss of signal, when comparing >7.2 *μ*M with <7.2 *μ*M Thimerosal, correlates well with changes in cell morphology, cell shrinkage and the formation of a ruffled plasma membrane and blebs.

In the time course of ethylmercury-induced changes shown in Figure 1(b), we see that the generation of ROS species is an early event and that there is an increase in ROS generation prior to changes in mitochondrial membrane potential. The levels of cellular DCF begin to fall at >40 minutes, and this drop in the levels of the cellular ROS reporter also correspond the observation of cell shrinkage and the formation of cytoplasmic blebs.

### 3.2. Colocalization of Mitotracker and ROS: Thimerosal Increases Mitochondrial Generation of ROS

**Figure 2:**
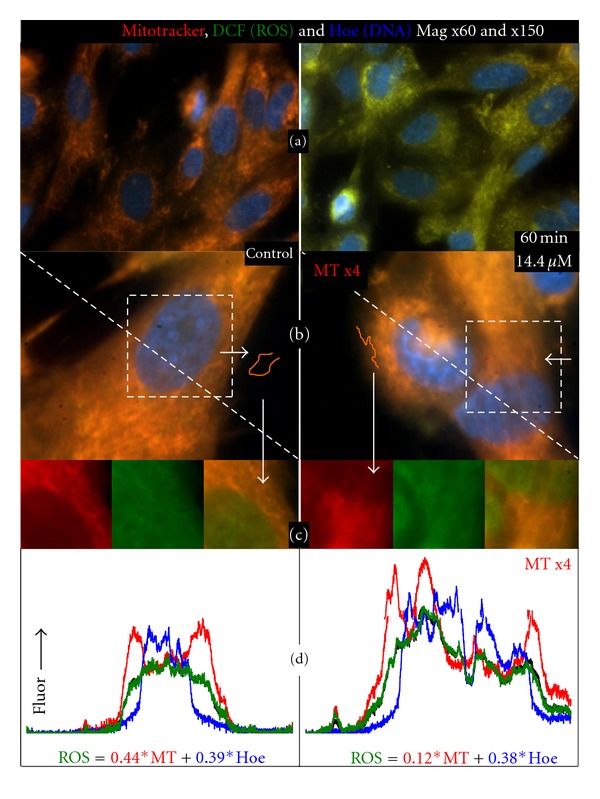
Colocalization of Mitotracker (ΔΨ) and DCF (peroxide) fluorescence in normal human astrocytes. Thimerosal induces oxidative stress at the mitochondrial level. High resolution images of control NHAs and NHAs treated for 60 minutes with 14.4 *μ*M Thimerosal. (a) Mitotracker (red), ROS-induced DCF (green), and nuclear Hoechst staining (blue) of NHAs at × 60 in the absence (left) and presence (right) of 14.4 *μ*M Thimerosal. (b) Images of control and treated cells obtained at × 150 magnification. An orange-colored “horseshoe” shaped signal in the control cell consists of a network of mitochondria which is mirrored in the ROS-induced DCF image. The same is demonstrated in the treated cells by a “lightening bolt” shaped mitochondrial network. (c) Square outlines of the cells from (b) highlighting individual Mitotracker and ROS images, and their overlaid images. (d) Intensity profile of MT, DCF, and Hoechst along the two diagonal lines in (b) (with the MT signal × 4 in the Thimerosal treated image). Red: MT signal, blue: Hoechst signal, green: ROS-generated DCF, black: fit to the ROS signal, based on the amplitudinal changes of MT and Hoechst. The two simulations indicate that four times the amount of DCF is generated by mitochondria in the ethylmercury-treated cells, but background cytosolic rates of generation are the same.

In Figure 2 we show the colocalization of mitochondrial and ROS signals in high resolution images of control NHA treated for 60 minutes with 14.4 *μ*M Thimerosal. In Figure 2(a), upper panels, we present the Mitotracker (red), ROS-induced DCF (green), and nuclear Hoechst staining (blue) of NHA taken at magnifications of × 60 in the absence (left) and presence (right) of 14.4 *μ*M Thimerosal. The fluorescence levels of all three panels are matched in the two images, so that the color levels absolutely reflect signal levels and show that Thimerosal causes an approximately 50% drop in mitochondrial membrane potential and a two-fold increase in ROS. It is clear that the majority of mitochondria in the cells are in a vermiform network and that there appears to be a strong colocalization of the mitochondrial and ROS signals.

In Figure 2(b) the images of control and treated cells obtained at × 150 magnification are shown. Here, the red mitochondrial signals are multiplied by a factor of four in the 14.4 *μ*M Thimerosal-treated astrocytes, so as to allow visual identification of the distribution of the mitochondria within these cells. The three treated cells shown are reasonably representative of the population with the central cell being shrunken and with a highly distorted nucleus. The square outlines are areas of the cells where we present individual Mitotracker and ROS images, and the overlaid images of these fluorophores of these chosen areas, Figure 2(c). These images clearly show that an orange colored “horseshoe” shaped signal in the control cell, shown in Figure 2(b), consists of a network of mitochondria and that this mitochondrial network is mirrored in the DCF, ROS, image. The correlation of mitochondrial signaling in the treated cells is also indicated, and in one of the treated cells we have identified a “lightening-bolt” shaped mitochondrial network. One can observe that this “lightening bolt” feature consists of mitotracker positive network of mitochondria and DCF signals.

Both images in Figure 2(b) have a diagonal line running from top-left to bottom-right. The bottom panel, Figure 2(d), shows the intensity profile of MT, DCF, and Hoechst along these two lines (with the MT signal × 4 in the Thimerosal treated image). The red lines correspond to the fluorescence signal of MT, the blue lines to Hoechst and the green lines to ROS generated DCF. In both plots there is an additional black line, which matches the line shape and amplitude to the DCF signal. This black line is our fit to the ROS signal, based on the amplitudinal changes of MT and Hoechst. In the control panel the ROS signal is best simulated by 0.44 multiplied by the MT signal and 0.39 multiplied by the Hoechst signal. In the Thimerosal-treated cells the relationship between Hoechst staining and DCF levels is within 3% of that in the control cells modeled at 0.38 multiplied by Hoechst fluorescence. However, the fit with MT labeling is strikingly different, with the best simulation generating a value of 0.117 for the ratio of actual MT signal to ROS. We compared the cross-correlation of the simulated fit with the actual DCF signal and found the slopes were 1 ± 0.01 in both cases and that the *R* ^2^ values were greater than 0.99 in both controls and treated cells. Thus, Thimerosal treated astrocytic mitochondria are generating four times the amount of ROS as the control mitochondria, but the steady state generation of ROS in areas with no mitochondria, especially the nucleus, is unchanged.

### 3.3. ROS Damage and Mitochondrial Membrane Potential

**Figure 3:**
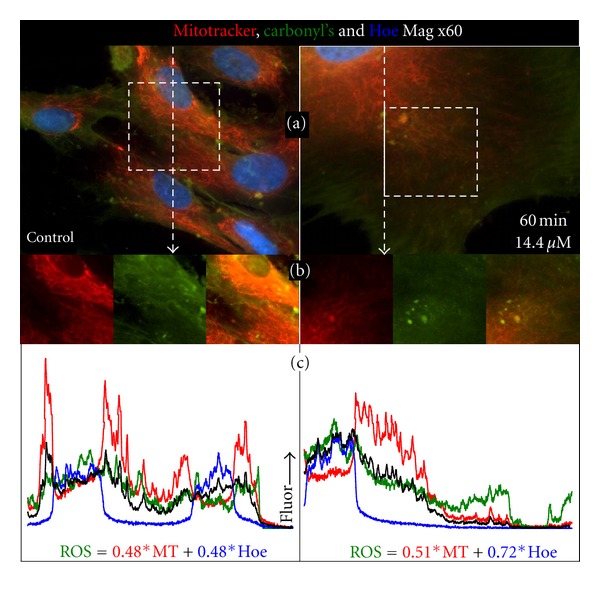
Colocalization of Mitotracker and carbonyls in normal human astrocytes. Thimerosal induces oxidative damage at the mitochondrial level. Control and 14.4 *μ*M Thimerosal-treated cells prepared using MT (red) and Hoechst (blue), with FITC-Avidin/Biotin-Hydrazide carbonyl labeling (green). (a) A large ethylmercury-treated cell showing an increase in green ROS damaged cell contents as a function of distance from the nucleus. (b) Two boxes from (a) highlighting the correlation between MT (red) and carbonyl (green) signals. (c) The two vertical lines in (a) indicate the position of fluorophore intensity profile interrogation. Red: MT, green: carbonyls, blue: Hoechst, black: a simulation of the levels of ROS damage generated by combining fractions of the MT and Hoechst signals. In both samples the simulation is a poor match for the actual ROS-induced signal, with cross-correlations of ROS versus simulation giving *R*_2_ values of only 0.68 and of 0.86, respectively.

In Figure 3 we show that damage from ROS, in the form of aldehyde/ketones (carbonyls), is also colocalized with mitochondrial membrane potential, and that more carbonyls are present in Thimerosol treated NHA. Figure 3(a) shows control and 14.4 *μ*M Thimerosal prepared using MT and Hoechst, then treated with Biotin-XX Hydrazide carbonyl labeling, which was visualized using FITC-Avidin.

Figure 3(a) shows control/Thimerosal-treated cells where all three fluorophores have the same scale. We chose to present the images of a large ethylmercury-treated cell, somewhat unrepresentative of the population size distribution, as larger cells allow easier discrimination of the mitochondrial network. What is noticeable is that there is an increase in green ROS damaged cell contents as a function of distance from the nucleus, in both images. The two boxes in Figure 3(a) show areas we have chosen to highlight the correlation between MT and carbonyl signals. These areas are shown as single images of MT and carbonyls, and as a merged image in Figure 3(b). In the control cells it is clear that the network of mitochondria is colocalized with some networks of carbonyls, but there are some well-defined structural networks which show evidence of oxidative stress that do not correlate with mitochondria. We observe a similar pattern in Thimerosal-treated astrocytes; there are quite clearly networks of mitochondria and carbonyls and structures that contain evidence of ROS damage, but without polarized mitochondria. The two vertical lines in Figure 3(a) indicate the position the fluorophores were interrogated to generate the fluorophore profiles shown in Figure 3(c). Again the three colors represent different fluorophores, MT (red), carbonyls (green) and Hoechst (blue), and the black line is a simulation of the levels of ROS damage generated by combining fractions of the MT and Hoechst signals. In both samples the simulation is a poor match for the actual ROS-induced signal, but control cells give a much poorer fit than do Thimerosal-treated astrocytes. Cross-correlations of ROS versus our simulation of carbonyl levels generate slopes of 0.75 and 1.1 for controls and ethylmercury-treated cells, and prove *R* ^2^ values of only 0.68 and of 0.86, respectively. Therefore, although we observe that generation of ROS is highly localized to mitochondria (Figure 2), the cellular distribution of markers of ROS damage is poorly localized with mitochondria.

It therefore appears that proteins suffering ROS damage, and so having carbonyls, are transported from the regions where they have been damaged. Vesicles containing high levels of carbonyls are present in both the controls and treated cells; however, in the cells that have been incubated with ethylmercury we observe a large number of small, <500 nm, clumps of oxidized material. A possible origin of this material is that it represents flocculated damaged mitochondria that are unable to maintain a membrane potential, such as that which occurs following the mitochondrial permeability transition [4]. This clumping of mitochondria has previously been described during the early stages of apoptosis and has shown to be a result of the activation of the BH3 domain of BAX [38].

### 3.4. Colocalization of ROS Damage and mtDNA Damage: Thimerosal Attacks mtDNA

**Figure 4:**
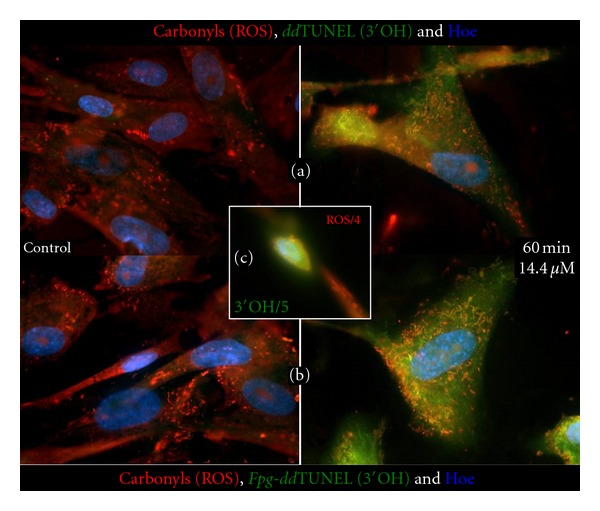
Colocalization of carbonyls and mtDNA damage normal human astrocytes. Thimerosal-induced oxidative damage occurs at mitochondrial matrix level. Nuclei (blue), carbonyls (red), and 3′OH DNA ends (green) in (a) and *Fpg*-labile DNA bases/apurinic and apyrimidinic sites (green) in (b). (c) (insert): A cell that has entered the final stages of apoptosis with large numbers of 3′OH DNA ends derived from nuclear DNA.

We initially postulated that cationic, lipophilic ethylmercury should partition into the mitochondrial matrix and that accumulation should be driven by the mitochondrial membrane potential. As mtDNA is restricted to the mitochondrial matrix, an increase in the steady state of ROS in this compartment should act as a reporter of this oxidative stress. We examined the presence of 3′OH DNA breaks or of *Fpg*-labile modified DNA bases using *dd*TUNEL and *Fpg*-*dd*TUNEL [35], and additional aldehyde/ketones (carbonyls) using Biotin-XX Hydrazide. Cells grown in the absence or presence of 14.4 *μ*M Thimerosal were labeled for the presence of 3′OH DNA nicks (*dd*TUNEL) or for oxidized/acylated DNA bases (*Fpg-dd*TUNEL) using biotinylated *dd*UTP. These DNA ends were visualized using FITC-Avidin and carbonyls with Texas Red-Avidin, and nuclei were again labeled with Hoechst, Figure 4. The signals in Figure 4 show (blue) nuclei, (red) carbonyls, and (green) 3′OH DNA ends (Figure 4(a)) or (green) *Fpg*-labile DNA bases/apurinic or apyrimidinic sites (Figure 4(b)), reflecting the levels of fluorophore in all four images. The insert, Figure 4(c), is of a cell that has entered the final stages of apoptosis and so shows large numbers of 3′OH DNA ends. In Figure 4(c) the ROS signal has been divided by a factor of four and the *dd*TUNEL signal by a factor of five, compared to Figure 4(a) and (b). Comparison of Figure 4(c) with 4(a) and 4(b) reveals that at one hour of incubation we are not observing full blown apoptosis, which is characterized by nuclear DNA fragmentation, and are indeed observing the early phases of cell death.

What Figure 4 demonstrates is that there is a clear colocalization of DNA damage and the presence of carbonyls. The damaged DNA is cytosolic, not nuclear, suggesting mitochondrial DNA damage. By demonstrating a colocalization of mitochondrial DNA damage and ROS in the cytosol of the NHAs, we show that the mitochondria may be responsible for the generation of ROS in the presence of ethylmercury and are the primary inducers of the apoptotic cascade.

### 3.5. The Identity of the Oxidant Produced by Ethylmercury in Mitochondria

**Figure 5:**
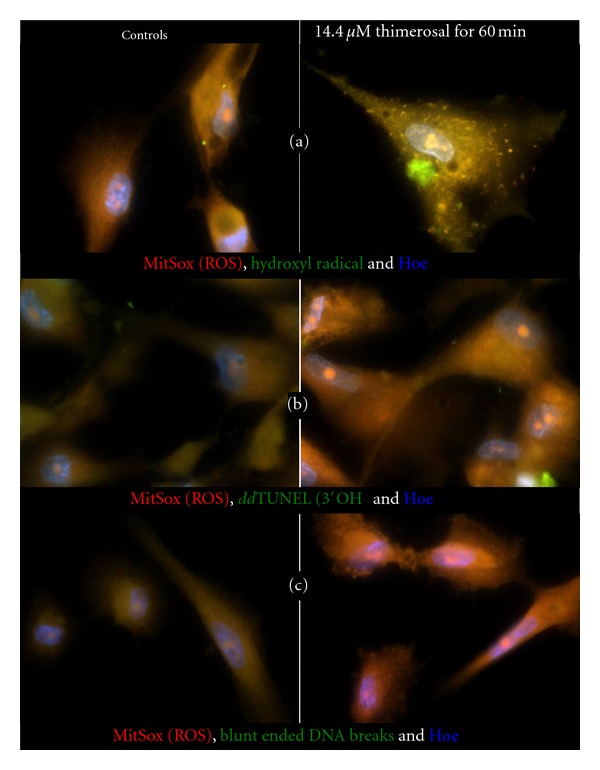
Mitochondrial superoxide production correlates with hydroxyl radical generation and mtDNA damage in normal human astrocytes. Thimerosal potentiates Fenton/Haber-Weiss chemistry in the mitochondrial matrix. Control NHAs and NHAs incubated for 1 hour with 14.4 *μ*M Thimerosal. Production of ROS measured with the mitochondrial superoxide probe MitoSox (red), and measurement of HO^•^ via hydroxyphenyl fluorescein (HPF) (green) in (a), 3′OH DNA ends with *dd*TUNEL (green) in (b), and blunt-ended DNA breaks (green) in (c).

We measured the production of ROS, using the mitochondrial superoxide probe MitoSox, and additionally measured HO^•^ via hydroxyphenyl fluorescein (HPF), 3′OH DNA ends with *dd*TUNEL and blunt-ended DNA breaks in NHA incubated for 1 hour with 14.4 *μ*M Thimerosal, Figure 5. Figure 5(a) shows that reporters for both superoxide and HO^•^ are highly colocalized, giving *R* ^2^ values of >0.98, and thus superoxide generation leads to Fenton/Haber-Weiss chemistry inside mitochondria. Treatment of NHA with ethylmercury leads to a 90% increase in superoxide generation per cell, even though under the same conditions we observe a 50% drop in mitochondrial membrane potential. Deconvolution of superoxide and HO^•^ signals show that the presence of ethylmercury results in 60% more HO^•^ generation per superoxide. Figures 5(b) and 5(c) show that superoxide generation correlates with nonnuclear, thus mtDNA damage in the form of *dd*TUNEL 3′OH DNA ends and also of highly damaging blunt-ended DNA breaks [36]. The scaling of the two green channels in Figures 5(b) and 5(c) differ by a factor of 4, and this indicates that there are on average 9 times as many 3′OH ends as there are DNA breaks in the control mitochondria.

### 3.6. Global Changes in Mitochondrial Function and Cellular Damage to NHA Resulting from Exposure to Ethylmercury

**Figure 6:**
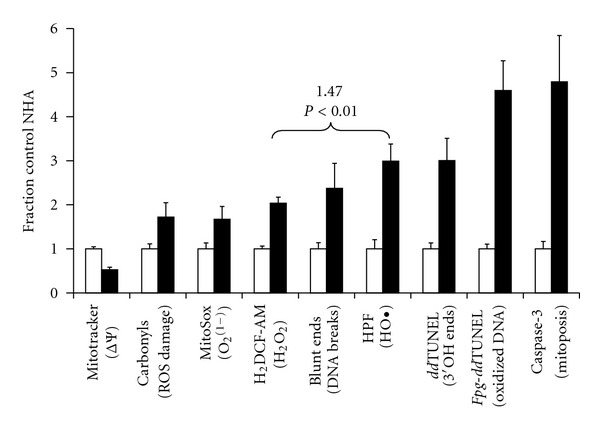
Summary of observed changes in NHA following incubation with ethylmercury. Bar plot showing the summarized changes observed in NHA following a one-hour exposure to 14.4 *μ*M Thimerosal, with respect to untreated controls. Each value is expressed as mean ± SD of five fields measured in the center field of triplicate experiments. Each of the treated values is statistically different from the controls at *P* < 0.01. The increase in HO^•^ is statistically different from the increase in H_2_O_2_ (H_2_DCF-AM) at *P* < 0.01. Statistical analyses were performed using one-way analysis of variance (ANOVA) with the Holm-Bonferroni post hoc test. The test was performed only when the results of ANOVA were *P* < 0.05, using Daniel's XL Toolbox, a free, open source add-in for Microsoft Excel.

In Figure 6 we present a bar plot showing the summarized changes we observe in NHA following a one-hour exposure to 14.4 *μ*M Thimerosal. All plots represent the average signal levels with respect to control cells. Five images were taken from three parallel experiments with an average of 44 ± 18 individual astrocytes per visual field, and the error bars represent the SD of the population.

Ethylmercury causes a 50% collapse in membrane potential in astrocytes at 1 hour. Accompanying this collapse in membrane potential we observe a significant increase in the levels of various ROS. The internal mitochondrial steady state level of superoxide increases by ≈70% in treated cells and is matched by an increase in cellular hydrazine reactive carbonyls. Using H_2_DCF-AM we observe a 200% increase in steady state production of reactive oxidants, which from deconvolution we know to be mitochondrially generated (Figure 2). Mitochondrial DNA, and not nuclear DNA, is far more vulnerable to ethylmercury-induced damage. We observe a 240% increase in the levels of mitochondrial DNA breaks, a 300% increase in 3′OH DNA nicks and 460% increase in the levels of oxidized bases/apurinic or apyrimidinic sites. As mtDNA is localized within the mitochondrial matrix, it follows that this is the main site of ROS generation. The 300% increase in HO^•^ is ≈80% greater than the increase in superoxide generation. As Fenton/Haber-Weiss chemistry is the primary generator of HO^•^ in biological systems [5], this finding suggests that ethylmercury is also increasing the levels of Fenton metals, such as iron and copper, inside the astrocytes' mitochondria.

In the final pair of bars were shown the change in the levels of Caspase-3 activity, measured by examining the cleavage of a *Z*-DEVD-R110 substrate. We also find a five-fold increase in Caspase-3 activity, indicating that this pathway has been activated in Thimerosal-treated cells.

## 4. Discussion

We find that treatment of NHA with ethylmercury causes an increase in mitochondrial superoxide generation as shown in Figure 5. However, the increase in superoxide generation is identical to the increase in the levels of protein carbonyls as shown in Figure 6. H_2_O_2_-induced formation of dichlorofluoresein from H_2_DCF-AM is only approximately 20% greater than superoxide/carbonyl formation, which suggests that the loss of peroxidase function is not a feature of NHA ethylmercury toxicity. This is consistent with the effect of methylmercury on HeLa cells, where mitochondrial matrix generation of superoxide was implicated as the most damaging ROS [39]. HeLa cells can be protected from methylmercury toxicity by upregulating mitochondrial Mn-SOD but not cytosolic Cu/Zn-SOD, GPx or catalase.

The majority of protein carbonyls in controls and in ethylmercury-treated NHA are also colocalized with mitochondria, as shown in Figure 2. The peroxides measured via H_2_DCF-AM and protein carbonyls are derived from mitochondrial ROS generation, as shown by colocalization of signals with the specific mitochondrial superoxide probe, MitoSox, as shown in Figure 5. These findings are in broad agreement with the known generation of ROS on either side of the inner mitochondrial membrane in normal mitochondria [40] and effects of methylmercury on rodent astrocytes observed by Shanker and coworkers [30], as they too identified that mitochondria are the main production sites of increased superoxide generation.

In addition to measuring peroxide/superoxide generation we also examined the formation of HO^•^ using the specific probe HPR and using the *Fpg*-*dd*TUNEL assay which measures oxidized DNA bases. The conversion of guanine to 8-hydroxyguanine and 8-hydroxyguanine to more oxidized DNA hydantoin lesions, spiroiminodihydantoin and guanidinohydantoin, is generally believed to be due to HO^•^ or to Fenton's reagent (oxy-ferry; Fe^(IV)^  =  O^(2−)^) and oxy-cupryl Cu^(III)^ = O^(2−)^) [41].

8-hydroxyguanine, spiroiminodihydantoin, and guanidinohydantoin are substrates from the *Fpg*-*dd*TUNEL assay [35, 42]. In Figure 4 we demonstrated that while the levels of damaged nuclear DNA and mtDNA are very low in untreated cells, ethlymercury induces a large increase in oxidized mtDNA lesions. The highest levels of damaged mtDNA and protein carbonyls occur in structures that appear to be flocculated mitochondria. These grainy, oxidized, structures are not present as bright grains when viewed using Mitotracker, when carbonyl rich grains can be identified, shown in Figure 2. These same vermiform structures are also identified in treated cells labeled with specific probes for both superoxide and HO^•^ seen in Figure 5. However, although we observe an increase in the levels of cytosolic (hence mitochondrial) blunt-ended breaks and nicks in Figure 5, very high levels of DNA breaks are not present in granular form. Thus, these flocculated mitochondria represent a dead-end mitochondrial state and given the close correlation between *Fpg*-*dd*TUNEL and Caspase-3 upregulation shown in Figure 6, it is reasonable to conclude these are mitochondria that have undergone the permeability transition [20], resulting in the release of proapoptotic proteins like cytochrome *c* and DIABLO from the intermembrane space, mitoposis, and the initiation of the Caspase-3 apoptotic cascade [4].

### 4.1. The Mechanism of Superoxide, Peroxide, and HO^•^ Generation in NHA

It has long been known that organomercury reacts with iron sulfur centers [43]; indeed methylmercury has been used as an aid to identify mercury adducts in iron-sulfur protein crystal structures for decades. The reaction of organomercury with iron sulfur centers in proteins such as aconitase results in loss of enzymatic function, the formation organomercury thioether adducts, and exposure to the bulks aqueous phase to redox active iron or release of free iron. It has been shown that, in mouse brain, the mitochondrial iron-sulfur complex rich enzyme NADH/Quinone oxidoreductase (Complex I) is highly sensitive to methylmercury [44]. In a study by LeBel et al. it was found that methylmercury neurotoxicity was partially iron mediated [37]. The potent iron-chelator, Deferoxamine, protected rat cerebellum from ROS following an injection of methylmercury. Iron chelation also protected neurons from ROS following *in* *vitro* exposure to methylmercury, but there was no evidence of deferoxamine-mercurial complex formation [37]. Methylmercury treatment of isolated mitochondria, from the cerebrum, the cerebellum and from liver, causes an inhibition of respiration and increased superoxide/hydrogen peroxide formation [29], mostly via damage to succinate dehydrogenase. The three iron-sulfur centers of succinate dehydrogenase on the matrix side of the inner mitochondrial membrane are the likely site of inhibition and possible iron release given that these clusters are sulphide/iron labile towards the thiophilic reagent, *p*-chloromercuribenzoate [45].

**Figure 7:**
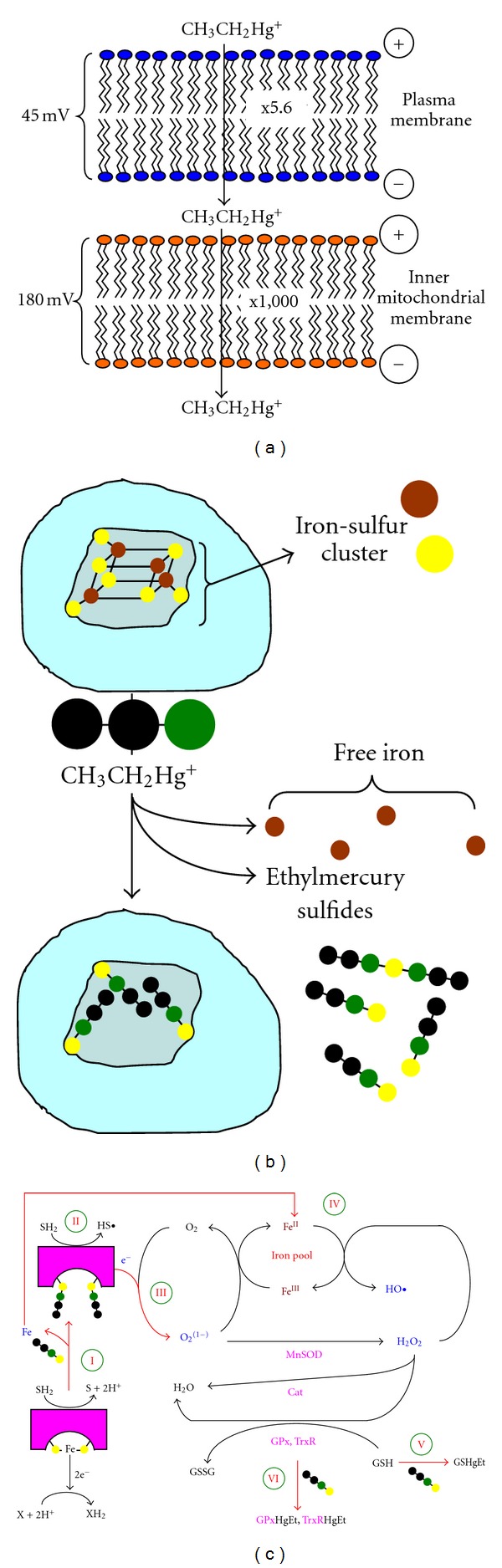
Proposed mechanism for the toxicity of organomercury. (a) As a lipophilic cation, ethylmercury will become concentrated inside astrocytes, following the plasma membrane potential of 45 mV [37], by a factor of 5.6 fold, and cytosolic ethlymercury will partition into the mitochondria by a factor of 1,000 fold, its accumulation driven by the approximate 180 mV mitochondrial membrane potential [25]. (b) Inside the mitochondria ethylmercury will react and cap thiols/selenols, including the cysteine residues of iron-sulfur centers. The formation of ethylmercuricthiol adducts will not only cause enzyme inhibition, but also increase the levels of free iron inside the mitochondria. (c) The release of iron catalyzes Fenton/Haber-Weiss chemistry leading to the formation of the highly oxidizing HO^•^. HO^•^ has multiple targets, including sensors of the permeability transition complex and also mtDNA. High levels of HO^•^ cause Mitoposis, leading to cytochrome *c* release from the mitochondria and the initiation of apoptosis.

Based on the work reported here and by others, we suggest a mechanism for the toxicity of organomercury, which is shown in diagrammatic form in Figure 7. As a lipophilic cation, ethylmercury will become concentrated inside astrocytes, with respect to the bulk extracellular phase, following the plasma membrane potential of 45 mV [46] by a factor of 5.6 fold, and cytosolic ethlymercury will partition into the mitochondria by a factor of 1,000 fold, its accumulation driven by the approximate 180 mV mitochondrial membrane potential [25], Figure 7(a).

Inside the mitochondria the ethylmercury will react with iron-sulfur centers, causing the release of iron into the mitochondrial matrix, Figure 7(b).

The role of ethlymercury in ROS species formation and detoxification is shown in Figure 7(c). The iron-sulfur centers of oxidoreductases (e.g., succinate dehydrogenase) when damaged by organomercury not only generate free iron, (Figure 7(b) I), but also form intraenzyatic carbon radical species (Figure 7(b) II) that will react with molecular oxygen to give rise to superoxide, (Figure 7(b) III). Superoxide can react with either free iron generation, the ferrous ion, or be dismutated into hydrogen peroxide by the mitochondrial Mn-SOD. Ferrous ion, and hydrogen peroxide react to generate the highly oxidizing radical, hydroxyl radical, (Figure 7(b) IV), an agent implicated in pathology and ageing [47, 48]. The levels of hydrogen peroxide would be generally lowered by the mitochondrial antioxidants, including glutathione-dependent selenol/thio-based peroxidases, like GPx and TrxR. However, these enzymes are inhibited by organomercury indirectly by depletion of glutathione, (Figure 7(b) V), and directly by the capping of the active site selenol/thiol by organomercury, (Figure 7(b) VI).

Thus, the release of iron catalyzes Fenton/Haber-Weiss chemistry leading to the formation of the highly oxidizing HO^•^. HO^•^ has multiple targets, including sensors of the permeability transition complex and also mtDNA. High levels of HO^•^ cause Mitoposis, leading to cytochrome *c* release from the mitochondria and the initiation of apoptosis. We find that a consequence of ethylmercury exposure to NHA is damage to the mitochondrial genome. We find an increase in DNA nicks, breaks and most importantly, in the level of oxidized bases. Mitochondria typically have 150 copies of mtDNA and during aging or with exposure to environmental stressors, the number of error free copies of mtDNA undergoes a decline. According to Harman's free radical/mitochondrial theory of aging [47, 48], the production of ROS by mitochondria leads to mtDNA damage and mutations. These in turn lead to progressive respiratory chain deficits, which result in yet more ROS production, producing a positive feedback loop.

The results of this study suggest that ethylmercury is a mitochondrial toxin in human astrocytes. We believe that this finding is important, particularly since the number of diseases in which mitochondrial dysfunction has been implicated are rapidly increasing.
